# Substrate Specificity of Cysteine Proteases Beyond the S_2_ Pocket: Mutagenesis and Molecular Dynamics Investigation of *Fasciola hepatica* Cathepsins L

**DOI:** 10.3389/fmolb.2018.00040

**Published:** 2018-04-19

**Authors:** Ileana Corvo, Florencia Ferraro, Alicia Merlino, Kathrin Zuberbühler, Anthony J. O'Donoghue, Lucía Pastro, Natalia Pi-Denis, Tatiana Basika, Leda Roche, James H. McKerrow, Charles S. Craik, Conor R. Caffrey, José F. Tort

**Affiliations:** ^1^Departamento de Genética, Facultad de Medicina, Universidad de la República, Montevideo, Uruguay; ^2^Laboratorio de Química Teórica y Computacional, Facultad de Ciencias, Universidad de la República, Montevideo, Uruguay; ^3^Department of Pharmaceutical Chemistry, Pharmacology, Biochemistry and Biophysics, University of California, San Francisco, San Francisco, CA, United States; ^4^Skaggs School of Pharmacy and Pharmaceutical Sciences, University of California, San Diego, San Diego, CA, United States; ^5^Departamento de Biología Celular y Molecular, Unidad de Biología Parasitaria, Facultad de Ciencias, Instituto de Higiene, Universidad de la República, Montevideo, Uruguay; ^6^Department of Pathology, Center for Discovery and Innovation in Parasitic Diseases, University of California, San Francisco, San Francisco, CA, United States

**Keywords:** *Fasciola hepatica*, cathepsin L, active site conformation, S2 pocket, mutagenesis, molecular dynamics simulation

## Abstract

Cysteine proteases are widespread in all life kingdoms, being central to diverse physiological processes based on a broad range of substrate specificity. Paralogous *Fasciola hepatica* cathepsin L proteases are essential to parasite invasion, tissue migration and reproduction. In spite of similarities in their overall sequence and structure, these enzymes often exhibit different substrate specificity. These preferences are principally determined by the amino acid composition of the active site's S_2_ subsite (pocket) of the enzyme that interacts with the substrate P_2_ residue (Schetcher and Berger nomenclature). Although secreted *Fh*CL1 accommodates aliphatic residues in the S_2_ pocket, *Fh*CL2 is also efficient in cleaving proline in that position. To understand these differences, we engineered the *Fh*CL1 S_2_ subsite at three amino acid positions to render it identical to that present in *Fh*CL2. The substitutions did not produce the expected increment in proline accommodation in P_2._ Rather, they decreased the enzyme's catalytic efficiency toward synthetic peptides. Nonetheless, a change in the P_3_ specificity was associated with the mutation of Leu67 to Tyr, a hinge residue between the S_2_ and S_3_ subsites that contributes to the accommodation of Gly in S_3_. Molecular dynamic simulations highlighted changes in the spatial distribution and secondary structure of the S_2_ and S_3_ pockets of the mutant *Fh*CL1 enzymes. The reduced affinity and catalytic efficiency of the mutant enzymes may be due to a narrowing of the active site cleft that hinders the accommodation of substrates. Because the variations in the enzymatic activity measured could not be exclusively allocated to those residues lining the active site, other more external positions might modulate enzyme conformation, and, therefore, catalytic activity.

## Introduction

*Fasciola hepatica* causes fascioliasis, a zoonotic disease that affects mainly cattle and sheep, and generates major economic loses worldwide. The World Health Organization classified it as an emerging neglected disease of humans (Keiser and Utzinger, 2009). There is an urgent need to develop new control strategies particularly with the increasing number of reports of resistance to the drug of choice, triclabendazole, in both livestock and humans (Cabada et al., 2016; Kelley et al., 2016). As in other parasites, liver fluke secreted proteases help colonize the mammalian host, and contribute to parasite development, survival and reproduction (Dalton et al., 2006; Robinson et al., 2009; Cancela et al., 2010). Papain-like cysteine proteases predominate and comprise an expanded multigenic family recently reported to have at least 14 distinct isoforms (McNulty et al., 2017). Among these, cathepsin L1 (*Fh*CL1) and to a lesser extent- cathepsin L2 (*Fh*CL2) are the most abundant enzymes secreted by adult flukes (Robinson et al., 2008, 2009). Despite their high sequence identity and overall structure conservation, *Fh*CL1 and *Fh*CL2 show distinct substrate specificities, highlighting that they might play different roles in parasite biology. The most notable difference is the ability of *Fh*CL2 to hydrolyze peptides with Pro in the P_2_ position (Dowd et al., 1997; Stack et al., 2008), an activity similar to that of human cathepsin K (Choe et al., 2006). This property confers on *Fh*CL2 the ability to cleave native collagen (Lecaille et al., 2008). A third collagen-digesting cathepsin, *Fh*CL3, is expressed exclusively in the invasive stage and might mediate the invasion process (Cancela et al., 2008; Corvo et al., 2009, 2013). Irving et al. identified residues with positive selection in the *Fasciola* cathepsin family, several of which are in the active site cleft and which might account for the different specificities of the enzymes (Irving et al., 2003).

**Figure 1:**
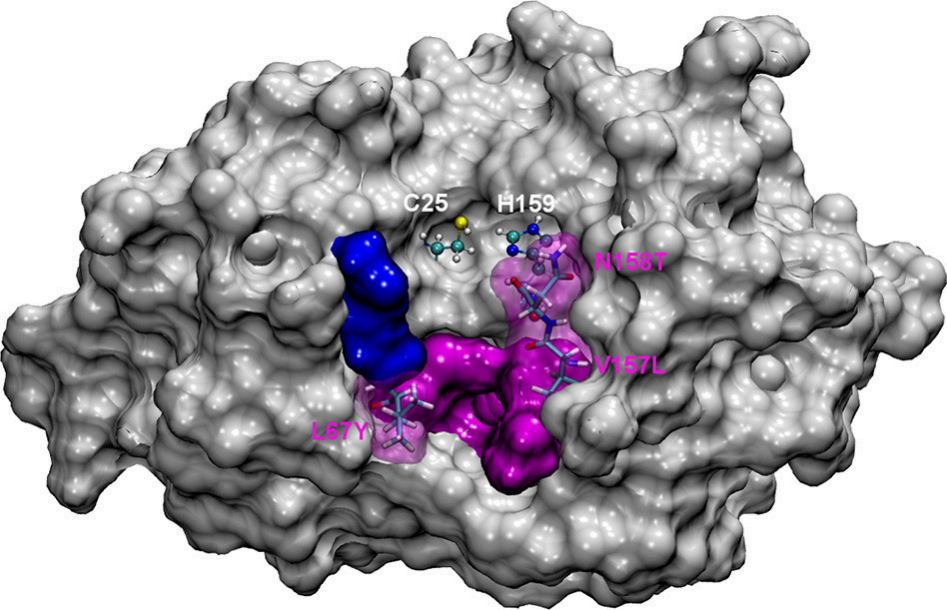
The most representative structure for *Fh*CL1 is shown in gray surface, the catalytic diad Cys25 and His159 is labeled and shown in balls and sticks; pink surface represents the S_2_ subsite and blue surface represents the S_3_ subsite. Residues mutated in this work are represented in sticks and labeled.

The general mechanism of catalysis of cysteine proteases relies on the nucleophilic attack of the peptide bond of the substrate by a catalytic Cys residue in the active site. The substrate specificity of the enzyme on the other hand is primarily determined by the interactions between the side chains of the residue of the substrate that is accommodated in the S_2_ subsite of the enzyme active site pocket (Schechter and Berger, 1967; Turk et al., 1998). Previous studies pointed to a role for the particular residues lining the S_2_ pocket (Table 1 and Figure 1) in conferring specific enzymes with their distinct substrate specificities (Pauly et al., 2003; Choe et al., 2006; Corvo et al., 2013). For example, positions 67 and 205 (papain numbering), respectively situated at the entrance and bottom of the S_2_ pocket, have an important contribution to the substrate specificities of human cathepsin K and L. The replacement of these residues in human cathepsin K with those present in human cathepsin L render the S_2_ preferences for short peptides similar to cathepsin L. Likewise, the converse experiment bestows cathepsin K-like specificities on cathepsin L (Lecaille et al., 2007). In *Fasciola*, both *Fh*CL1 and CL2 have a Leu205 residue but they differ at position 67 where *Fh*CL1 has Leu and *Fh*CL2 has Tyr. However, when *Fh*CL1 Leu67 was replaced with Tyr (as in *Fh*CL2), the ability of the mutated enzyme to cleave peptides with Pro in P_2_ was not significantly increased (Stack et al., 2008). Interestingly, the same substitution in *Fasciola* cathepsin L5 (a minor enzyme with substrate preferences similar to *Fh*CL1) was reported to increase its acceptance of peptides containing Pro at P_2_ (Smooker et al., 2000). Recently, we showed that the mutation of *Fh*CL1 Leu67 to Trp greatly increases its ability to accommodate Gly in S_3_, making this pocket similar to that of *Fh*CL2 and *Fh*CL3 (Corvo et al., 2013), and suggesting that the ability of *Fh*CL5 (Tyr67) to accommodate the Gly-Pro-Arg peptide was due to a better acceptance of Gly in P_3_.

**Table 1:** Residues found in the S_2_ pocket of human cathepsin L and K (*Hs*CatL, *Hs*CatK) and adult *F. hepatica* CLs.

**Protease**	**S**_**2**_ **pocket residues**				
	**67**	**157**	**158**	**160**	**205**
*Hs*CatL	Leu	Met	Asp	Gly	Ala
*Hs*CatK	Tyr	Leu	Asn	Ala	Leu
*Fh*CL1	***Leu***	***Val***	***Asn***	Ala	Leu
*Fh*CL2	***Tyr***	***Leu***	***Thr***	Ala	Leu
*Fh*CL5	Leu	Leu	Asn	Gly	Leu

*The residues mutated in this work are shown in bold italics. Residues were numbered according to papain numbering*.

The contribution of the other S_2_ residues, however, has not been assessed. Leu157 in *Fh*CL2, which is opposite to the gatekeeper residue Tyr67, was suggested to contribute to P_2_-Pro interaction by stabilizing the planar ring of Pro (Stack et al., 2008). Also, Thr158 in *Fh*CL2, which is located beside the catalytic His159, was proposed to influence the accessibility to the S_2_ pocket (Stack et al., 2008). Predictions of per-residue free energy decomposition studies on a *Fh*CL3 enzyme model provided further theoretical support for the contribution of these positions (67 and 157) to substrate binding (Hernández Alvarez et al., 2015). Val157 in *Fh*CL1 is a residue one carbon shorter than the Leu found in *Fh*CL2 and human cathepsin K. This is too short to contribute to aliphatic interactions and led Stack et al. to suggest that the lack of both the Tyr67 and Leu157 gatekeeper residues might account for the reduced preference for P_2_ Pro by *Fh*CL1 (Stack et al., 2008).

To study how the S_2_ pocket residues that differ between *Fh*CL1 and *Fh*CL2 contribute to substrate specificity, we engineered the *Fh*CL1 S_2_ pocket by replacing one or more of the Leu67, Val157 and Asn158 residues in the S_2_ pocket (Table 1 and Figure 1), with the respective residues present in *Fh*CL2. We analyzed the mutated enzymes by following the hydrolysis of synthetic fluorogenic peptides and by Positional Scanning- Synthetic Combinatorial Libraries of fluorogenic tetrapeptides (PS-SCL) (Choe et al., 2006). To gain further insights into the mechanisms of specificity, we performed molecular dynamic simulations on both native and mutant enzymes. Our results point to subtle differences in the secondary structure and geometry of the *Fh*CL1 mutants that hinder substrate accommodation and might underlie their reduced affinity and catalytic efficiency. The data introduce novel aspects to consider for the rational design of cysteine protease inhibitors which have shown therapeutic utility in the control of trematode infections (Abdulla et al., 2007; Ferraro et al., 2016).

## Materials and methods

### Generation of *Fh*CL1 mutants

Seven *Fh*CL1 variants bearing substitutions at the S_2_ active site pocket were generated by site-specific mutagenesis using the QuikChange Site-Directed Mutagenesis Kit (Invitrogen). Briefly, different pairs of complementary oligonucleotides containing the base pair substitutions to be introduced in the cathepsin gene sequences were used in a PCR reaction, using as template *Fh*CL1 cloned in the X4-Mfα-ScPas3 expression plasmid (kindly provided by Dr. R.J.S. Baerends and Dr. J.A.K.W. Kiel, Molecular Cell Biology Lab, Groningen Biomolecular Sciences and Biotechnology Institute, University of Groningen, The Netherlands). Double and triple variants were obtained by using plasmids bearing the single (or double) mutations as templates, with the only exception of the *Fh*CL1 V157L/N158T double variant, where a pair of oligonucleotides containing both changes was used (Supplementary Table 1). The plasmids were propagated in bacteria and sequenced to verify the presence of the desired mutations. Then, they were electroporated in the *Hansenula polymorpha* yeast strain as previously described (Faber et al., 1994).

### Production of *Fh*CL1 and mutants in yeast

*Fh*CL1 recombinant proenzyme was produced in the yeast *Hansenula polymorpha* as previously described (Corvo et al., 2009). Briefly, yeast transformants were cultured in 500 ml YEPD broth at 37°C to an OD_600_ of 4–6, harvested by centrifugation at 2,000 g for 10 min and induced by resuspending in 50 ml of buffered minimal media (0.67% yeast nitrogen base, 0.1 M phosphate buffer, pH 6.0, 1% methanol) for 36 h at 30°C. Recombinant propeptidase was secreted to the culture media, and recovered by 20–30-fold concentration of culture supernatants by ultrafiltration with a 10 kDa cut-off membrane. The proenzyme was autocatalytically activated to the mature form by incubation for 2 h at 37°C in 0.1 M sodium citrate buffer (pH 5.0) containing 2 mM DTT and 2.5 mM EDTA, then dialyzed against PBS, pH 7.3, and stored at −20°C. The protein concentration was assessed by the BCA method (Smith et al., 1985) and the amount of active enzyme was determined by titration against the specific cysteine protease inhibitor E-64c. The mutant enzymes were obtained using the same protocol for the production of *Fh*CL1.

### Multiplex substrate profiling by mass spectrometry (MSP-MS)

The enzymatic activity of *Fh*CL1 and *Fh*CL2 was tested by MSP-MS, a procedure designed for unbiased profiling of protease activity consisting of the cleavage of a library of unmodified tetradecapeptides followed by mass spectrometry identification of all cleavage products (O'Donoghue et al., 2012). Briefly, a highly diversified peptide library consisting of 228 synthetic tetradecapeptides containing all possible amino acid pairs, and near neighbor pairs was used to test enzymatic activity. All peptides had unmodified termini and consist of natural amino acids except Met that was substituted by norleucine and Cys omitted because of potential disulfide bond formation. The library was distributed into two pools consisting of 114 peptides and diluted to 1 μM in 0.1 M sodium phosphate, pH6.0, 1 mM DTT, 1 mM EDTA. An equal volume of *Fh*CL1 or *Fh*CL2 in the same buffer was added to the peptide pools such that the final concentration of the enzyme was 1 nM and 50 nM, respectively. The enzyme concentrations used for this experiment was determined based on the activity of *Fh*CL1 and *Fh*CL2 against fluorescent substrates. An enzyme-free assay was set up as a control. Assays were incubated at room temperature for a total of 4 h and a 25% of the reaction volume was removed after 60 min and 4 h and the enzyme quenched by addition of formic acid to a final concentration of 4%. Samples were desalted using C18 LTS tips (Rainin) and rehydrated using 0.2% formic acid prior to mass spectrometry acquisition. Peptide sequencing an LTQ Orbitrap-XL mass spectrometer (Thermo) under identical running conditions as outlined previously (Winter et al., 2017). Mass spectrometry peak lists were generated using in-house software called PAVA. To identify peptide cleavage products, data searches were performed against the library of 228 peptides using Protein Prospector software (http://prospector.ucsf.edu/prospector/mshome.htm, UCSF). Because, in general, substrate binding pockets beyond four residues on each side of the scissile bond do not contribute to substrate specificity, we focus on the amino acids present in the P4 to P4′ positions. These octapeptide (P4–P4′) cleavage products are provided as Supplemental Data Sheet. For database searching, tolerances of 20 ppm and 0.8 Da were used for parent and fragment ions, respectively. The following variable modifications were selected with a maximum of 2 modifications per peptide: amino acid oxidation (proline, tryptophan, and tyrosine) and N-terminal pyroglutamate conversion from glutamine. Protein Prospector score thresholds were set to 22 and 15 with maximum expectation values of 0.01 and 0.05 for protein and peptide matches, respectively. Peptide cleavage products were imported into iceLogo software v.1.2 to generate protease substrate specificity profiles (Colaert et al., 2009). Octapeptides (P4–P4′) corresponding to the peptide cleavage products were used as the positive dataset, and octapeptides corresponding to all possible cleavage sites in the 228-member library (*n* = 2.964) minus 154 sites that were discovered in the non-enzyme treated samples, to yield a total of 2,810, were used as the negative dataset (see Supplemental Data Sheet).

### Protease assays using synthetic fluorogenic peptides

Short fluorogenic peptides are a fast and simple method to measure protease enzymatic activity and are commonly used to study substrate specificity using peptides with different sequences. Here, the protease activity was monitored by the hydrolysis of the fluorophore 7-amino-4-methyl coumarin (AMC) from the synthetic peptide substrates Z-Val-Leu-Lys-AMC and Tos-Gly-Pro-Arg-AMC (Z and Tos correspond to Carboxybenzyl and Tosyl, respectively; the blocking groups that enable cathepsin endopeptidases to position for peptide hydrolisis). The kinetic parameters were determined in a reaction buffer containing 0.1 M sodium phosphate buffer, pH 6.0, 1 mM DTT and 1 mM EDTA at 25°C with final enzyme concentrations in the 10^−9^ M range. Different substrate concentrations (5–100 μM) were added after a 10 min pre-incubation of the enzyme in reaction buffer and reaction rates were measured in duplicate. The slope of the progress curves were obtained by continuous recording in a FluoStar spectrofluorimeter at 345 nm excitation and 440 nm emission wavelengths, using an AMC standard curve for product concentration calculation. The enzyme concentration was determined by active-site titration with E-64c. The kinetic parameters V_max_ and *K*_M_ were estimated by non-linear regression analysis of the Michaelis–Menten plot using the OriginPro 6.1 software. *k*_cat_ was calculated as V_max_/[E] where [E] is the active enzyme concentration (fit to the Michelis-Menten equation for *Fh*CL1 hydrolysis of Z-Val-Leu-Lys-AMC peptide is included as an example in Supplementary Table 2). The *Fh*CL2 recombinant enzyme for the kinetic analysis was kindly provided by Prof. John Dalton (School of Biological Sciences, Queen's University Belfast).

### P_1_-P_4_ specificity testing using a positional scanning—synthetic combinatorial library (PS-SCL)

The substrate specificities of the *Fh*CL1 and *Fh*CL1 S_2_ pocket mutants were studied using a Positional Scanning- Synthetic Combinatorial Library (PS-SCL). This is composed of synthetic peptides with the general structure of acetyl-P_4_-P_3_-P_2_-P_1_-ACC, a bifunctional fluorophore leaving group similar to AMC. It consists of four libraries (P_1_, P_2_, P_3_, and P_4_) in which one position P_1_, P_2_, P_3_ or P_4_ is fixed with one of the 20 amino acids (omitting cysteine and including norleucine), whereas the remaining three positions contain an equimolar mixture of all amino acids. Each library consists of 20 sub-libraries, one for each fixed amino acid containing 8.000 different compounds (Harris et al., 2000; Choe et al., 2006). Assays were performed in triplicate in 0.1 M sodium phosphate buffer pH 6.0, 1 mM DTT, 1 mM EDTA, 0,01% PEG-6000 and 0.5% Me_2_SO (from the substrates) at 25°C. Aliquots of 12.5 nmol in 0.5 μl from each of the 20 sub-libraries were added to the wells of a 96-well Microfluor-1 flat-bottom plates. The final concentration of each compound of the 8.000 compounds per well was 15.62 nM in a 100 μl final reaction volume. The reaction was started by addition of the enzyme diluted in the above buffer and monitored with a SpectraMax Gemini fluorescence spectrometer (Molecular Devices) with excitation at 380 nm, emission at 460 nm and cutoff at 435 nm.

### Homology modeling and molecular dynamic simulations

Homology models of *Fh*CL1 and *Fh*CL2 were generated with SwissModel (Arnold et al., 2006) using as the principal template the crystal structure of the pro-cathepsin *Fh*CL1 (PDB ID: 2O6X). Template and models were superimposed for visualization with Swiss PDBViewer version 4.1. (Guex and Peitsch, 1997; http://www.expasy.org/spdbv/). Active site residues were identified based on the literature and confirmed by structural alignment with human cathepsin L (PDB ID: 1MHW), human cathepsin K (PDB ID: 1ATK) and papain (PDB ID: 5PAD). The *Fh*CL1 mutants were generated with DS Visualizer. In order to improve structure accuracy, MD simulations were performed using the *pmemd* module implemented in the AMBER14 package (Case et al., 2014), with the *ff14SB* force field (Maier et al., 2015). Hydrogen atoms and sodium ions (to neutralize charge) were added to each protein with the *leap* utility. Each system was placed in a truncated octahedral box of TIP3P explicit water (Jorgensen et al., 1983) that was extended 12 Å outside the protein on all sides. The structures of *Fh*CL1, *Fh*CL2 and the *Fh*CL1 mutants were treated as follows: water and counter ions were relaxed over 2,500 steps (500 steepest descent steps, SD, and 2,000 conjugate-gradient steps, CG) with the protein restrained with a force constant of 500 kcal/molÅ^2^. Then, the systems were minimized without restraints during 20,000 steps (5,000 SD and 15,000 CG). The cutoff distance for direct calculation of non-bonded interactions was set to 10 Å. Beyond this distance, electrostatic interactions were calculated using the Particle-Mesh-Ewald (PME) method (Essmann et al., 1995). After minimization, each system was gradually heated in a NVT ensemble from 0 to 300 K over 100 ps using the Berendsen coupling algorithm (Berendsen et al., 1984). This procedure was followed by 50 ns of NPT simulations at 300 K and 1 atm pressure using the Monte Carlo barostate algorithm (Faller and De Pablo, 2002). All bonds involving hydrogen atoms were constrained using the SHAKE algorithm (Ryekaert et al., 1997). The equations of motion were integrated with a time step of 2.0 fs and coordinates of the systems were saved every 2 ps. Representative structures of *Fh*CL1, *Fh*CL1 mutants and *Fh*CL2 from the trajectories were obtained through cluster analysis using the average-linkage algorithm (Shao et al., 2007) and used for further inspection of the structures. Clustering, RMSD, RMSF, hydrogen bond and DSSP analysis were performed using the *cpptraj* module in AmberTools14. For trajectories, visualization and figures of this paper the VMD program was used (Humphrey et al., 1996).

## Results and discussion

### *Fh*CL1 and *Fh*CL2 multiple substrate profiling by mass spectrometry (MSP-MS)

**Figure 2:**
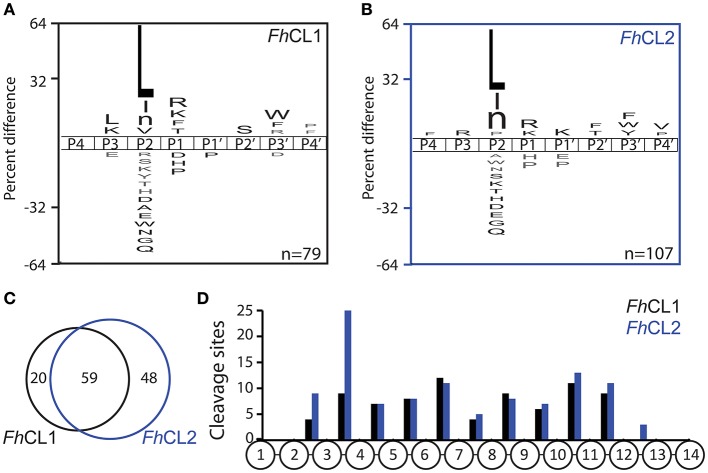
Differential cleavage specificities of *Fh*CL1 and *Fh*CL2 using MSP-MS. A. iceLogo plot illustrating the frequency of amino acids found in the P4-P4′ position of **(A)** *Fh*CL1 and **(B)** *Fh*CL2, following incubation with the 228-member peptide library for 4 h. Amino acids that are significantly enriched (top) or de-enriched (bottom) (*p* ≤ 0.05) are colored black. Lowercase “n” in the amino acid list corresponds to norleucine. The number of cleavage sites used to build the iceLogo plot is indicated in the right lower corner of the plot. **(C)** Venn diagram showing the number of shared and unique cleavage sites for *Fh*CL1 (black) and *Fh*CL2 (blue). **(D)** Distribution of the cleavage sites observed within the 14-mer peptides.

We employed the MSP-MS method to compare the specificity profile of the two main cathepsins of adult liver flukes. This method has been used to uncover the substrate specificity profile of proteases resulting in the development of optimized fluorescent substrates (O'Donoghue et al., 2015; Winter et al., 2017), peptide inhibitors (Li et al., 2016; Roncase et al., 2017), activity based probes (Lentz et al., 2016) and activity based biomarkers (Ivry et al., 2018). After 1 h incubation of each enzyme with the peptide library, *Fh*CL1 and *Fh*CL2 cleaved at 46 and 54 sites, respectively. Incubation for a further 3 h yielded 79 and 107 sites, respectively (Supplemental Data Sheet). We generated a substrate specificity profile using the P_4_ to ${\text{P}}_{4^{\text{′}}}$ amino acid frequency of the confirmed cleavage sites by comparing it to the amino acid frequency of all possible cleavage sites within the peptide library (n = 2.810). This profile confirmed that the S_2_-P_2_ interactions dominated the substrate specificity for both enzymes, as expected for clan CA cysteine proteases (Turk et al., 1998; Pauly et al., 2003; Figures 2A,B). Both enzymes preferentially cleaved at sites where Leu, Ile and norleucine were in the P_2_ position, however, *Fh*CL1 also cleaved when Val was in the P_2_ position, while *Fh*CL2 preferred Pro, consistent with previous reports (Dowd et al., 1997; Stack et al., 2008). Both enzymes share a preference for Lys and Arg at the P_1_ position and Trp and Phe at ${\text{P}}_{3^{\text{′}}}$. When the cleavages sites were directly compared, 59 of the sites were cut by both enzymes, however 48 sites were uniquely cleaved by *Fh*CL2 (Figure 2C). We next investigated the positon of cleaved within the 14-mer peptides and found that neither enzymes have aminopeptidase or carboxypeptidase activity since no peptide bonds in between residues 1 and 2 or between residues 13 and 14 were cleaved. Interestingly, many of the sites that are cleaved by *Fh*CL2 and not by *Fh*CL1 are found between residues 3 and 4, indicating that *Fh*CL2 may have some tripeptidyl-peptidase activity that is absent in *Fh*CL1 (Figure 2D).

### Kinetic analysis of the cathepsin mutants with short peptide substrates

To test the contribution of different active site residues involved in the enzymatic activity and amino acid preference of *Fh*CLs we engineered the *Fh*CL1 S_2_ pocket to resemble that of *Fh*CL2. The changes correspond to key residues at the entrance and wall of the S_2_ pocket that had been proposed by us and others to be important for substrate recognition (Table 1; Stack et al., 2008; Corvo et al., 2009, 2013). We then employed two different tripeptide substrates that contain Leu or Pro at P_2_ to evaluate the mutant specificities.

The fluorescent substrate Z-Val-Leu-Lys-AMC is hydrolyzed by *Fh*CL1 and *Fh*CL2 with similar efficiency, however, Tos-Gly-Pro-Arg-AMC is cleaved by *Fh*CL2 with >50-fold higher efficiency mostly due to a higher *k*_cat_ (Table 2). All the amino acid changes introduced result in a decrease in the hydrolysis of Z-Val-Leu-Lys-AMC, either due to a diminution of the *k*_cat_ (substitutions Leu67Tyr and Val157Leu), or a reduction in affinity associated with the Asn158Thr mutation (Table 2). These results suggest that the mutated *Fh*CL1 S_2_ subsite cannot interact properly with the substrate, or does it in an orientation that is unfavorable for catalysis.

**Table 2:** Kinetic parameters of *Fh*CL1, *Fh*CL2 and the mutant enzymes over two different substrates.

**Enzyme**	**Z-Val-Leu-Lys-AMC**			**Tos-Gly-Pro-Arg-AMC**		
	**k_cat_ (s^−1^)**	**K_M_ (μM)**	**k_cat_/K_M_ (M^−1^s^−1^)**	**k_cat_ (s^−1^)**	**K_M_ (μM)**	**k_cat_/K_M_ (M^−1^s^−1^)**
*Fh*CL1	2.32 ± 0.12	3.94 ± 0.60	5.89 × 10^5^	0.035 ± 0.006	15.10 ± 2.70	2.36 × 10^3^
*Fh*CL1 Leu67Tyr	0.53 ± 0.11	1.72 ± 0.06	3.08 × 10^5^	0.024 ± 0.003	8.52 ± 2.70	2.82 × 10^3^
*Fh*CL1 Val157Leu	0.72 ± 0.07	5.23 ± 0.58	1.38 × 10^5^	0.027 ± 0.001	13.16 ± 1.51	2.08 × 10^3^
*Fh*CL1 Asn158Thr	2.12 ± 0.14	18.38 ± 4.1	1.16 × 10^5^	0.023 ± 0.001	6.84 ± 1.73	3.32 × 10^3^
*Fh*CL1 Leu67Tyr Val157Leu	0.17 ± 0.010	3.85 ± 0.61	4.31 × 10^4^	0.027 ± 0.003	6.57 ± 2.40	4.01 × 10^3^
*Fh*CL1 Leu67Tyr Asn158Thr	1.35 ± 0.10	23.30 ± 4.00	5.78 × 10^4^	0.031 ± 0.004	8.81 ± 3.80	3.51 × 10^3^
*Fh*CL1 Val157Leu Asn158Thr	1.67 ± 0.11	10.2 ± 1.94	1.63 × 10^5^	0.015 ± 0.001	3.32 ± 1.90	4.52 × 10^3^
*Fh*CL1 Leu67Tyr Val157Leu Asn158Thr	0.65 ± 0.07	25.6 ± 6.7	2.54 × 10^4^	0.030 ± 0.002	6.86 ± 1.85	4.40 × 10^3^
*Fh*CL2	1.65 ± 0.24	4.72 ± 1.1	3.49 × 10^5^	0.720 ± 0.062	5.28 ± 2.01	1.36 × 10^5^

An increased affinity for Tos-Gly-Pro-Arg-AMC was generally observed in the mutants, but as the *k*_cat_ is slightly diminished, the specificity remains similar to that of *Fh*CL1. This behavior has also been observed by Stack et al. for the substitution Leu67Tyr in *Fh*CL1 (Stack et al., 2008). In the adult enzyme *Fh*CL5, however, the Leu67Tyr mutation provoked an increase in *k*_cat_ and activity toward Tos-GPR-AMC (3- to 10-fold as reported by Smooker et al., 2000; Norbury et al., 2012), an effect proposed to be due to the presence of the bulkier Leu at 157 that is also positioned at the entrance of the S_2_ pocket opposite residue 67. Yet, in our experiments, the substitution Val157Leu in *Fh*CL1, when combined with Leu67Tyr did not show a significant increase in specificity for Tos-Gly-Pro-Arg-AMC.

### P_2_ and P_3_ preferences of *Fh*CL1 mutants based on PS-SCL

**Figure 3:**
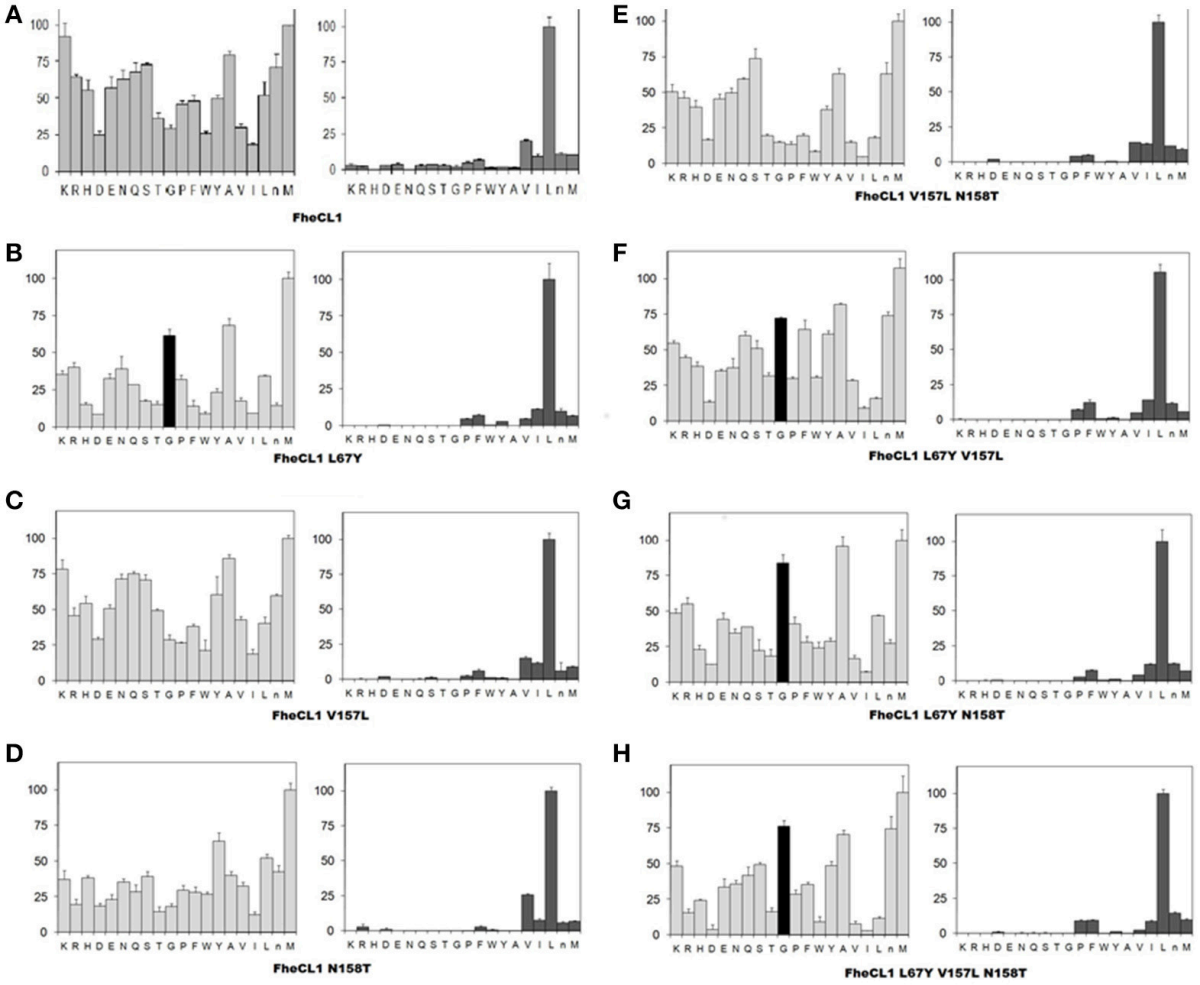
Profiling of the P_2_ and P_3_ substrate specificity of *Fh*CL1 enzyme variants using PS-SCL **(A–H)**. The y axis shows the enzymatic activity, against each of the 20 sub-libraries with P_2_ and P_3_ position of the substrates fixed, as a percentage relative to the highest activity in each library (the hydrolysis rates for the Leu- and Met- fixed peptide pools at P_2_ and P_3_, respectively, are taken as 100%). The x axis shows the different fixed amino acids using the one-letter code (*n* = norleucine). The error bars display the standard deviation from triplicate experiments.

A second step in the characterization of the mutant enzymes was assessing their individual amino acid preferences by PS-SCL (Choe et al., 2006). In Figure 3 we show the specificity profile of *Fh*CL1 and the mutant enzymes at the P_2_ and P_3_ position of the substrates. We found that neither of the S_2_ mutations alone or in combination could significantly modify the *Fh*CL1 preferences at P_2._ Even the triple mutant, in which the S_2_ pocket of *Fh*CL1 has exactly the same residues as *Fh*CL2, did not increase significantly its acceptance of Pro in that position (Figure 3H). However, the substitution Leu67Tyr increased the acceptance of Gly in P_3_ (black colored bar), both in the single change variant (Figure 3B) and in the double or triple substitutions (Figures 3F–H). We corroborated that the P_1_ preferences were not modified by any of the mutations (data not shown). These results suggest that even when the *Fh*CL1 residues lining the S_2_ pocket are identical to those in *Fh*CL2, the overall conformation of the subsite remains different as the *Fh*CL1 triple mutant cannot favorably interact with a P_2_ Pro. However, the S_3_ specificity of *Fh*CL2 can be mimicked in *Fh*CL1 when replacing Leu for Tyr at position 67, conferring it a preference for Gly that is similar to that observed in *Fh*CL2 and *Fh*CL3 (compare with Figure 3 of Stack et al., 2008; Corvo et al., 2013). In summary, the better accommodation of Gly in P_3_ as shown by the PS-SCL slightly improves the affinity of the *Fh*CL1 mutants bearing the Leu67Tyr substitution for substrates such as Tos-Gly-Pro-Arg-AMC but the interaction does not promote a more efficient hydrolysis of this peptide.

### Molecular dynamics of the active site configuration

**Figure 4:**
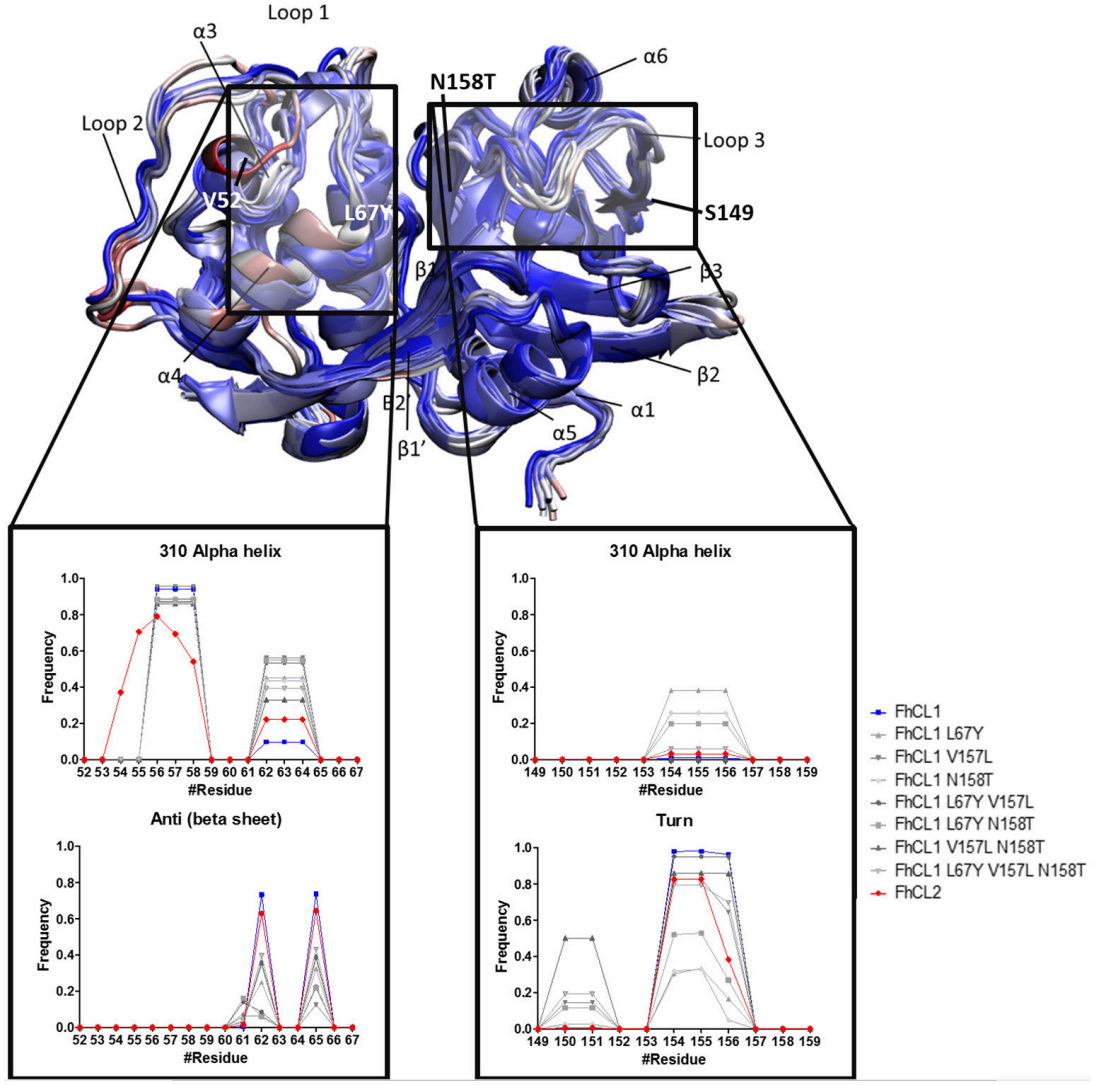
Standardized Definition of Secondary Structure Protein (DSSP) from the S_2_ and S_3_ subsites, and surrounding residues. Representative structures of *Fh*CL1, *Fh*CL2 and mutants are shown in new cartoons colored by RMSD of the alpha carbon by residue. Labels indicate main components of the enzyme structure and the rectangles mark the regions analyzed in the graphics. The graphics show the frequency values obtained from DSSP for residues 52–67 (S_2_ and its surroundings) and 149–159 (S_3_ and its surroundings).

To further investigate the active site determinants, we studied the differences in the overall conformation and the secondary structure between the representative structures of *Fh*CL1 and *Fh*CL2 obtained by hierarchical clustering. Then, we compared the structural features of the native enzymes with those of the *Fh*CL1 mutants. In order to improve structure accuracy we performed molecular dynamics simulations over the structures previously obtained by homology modeling (Corvo et al., 2013). We measured subtle changes in the protein backbones and minor structural variation between the enzymes particularly in the loops 1 and 3 which comprises residues 52–67 and 149–159 of the mature enzyme, respectively (Figure 4 and Supplementary Figure 1). A recent structural study of human cathepsin K and related papain-like proteases showed that loops edging the S_1_, S_2_, and S_3_ pockets of the active site are flexible (Novinec, 2017). In the *Fh*CL1 mutants, loop 1 adopts a 310 helix conformation with a higher frequency than the native enzymes (Figure 4). Similarly, on the other side of the active site, loop 3 also tends to form a 310 helical arrangement while reducing the turn configuration, changes that would result in a more rigid structure (Figure 4). Furthermore, a hydrogen bond, formed between residues Asn60–Gly65 in the S_3_ pocket of the *Fh*CL1 mutant enzymes, might contribute to forming the helical configuration seen in the proximities of this site. Taken together, our data suggest that minor conformational changes at the edges of the active site may be sufficient to modify these enzymes' activity.

**Figure 5:**
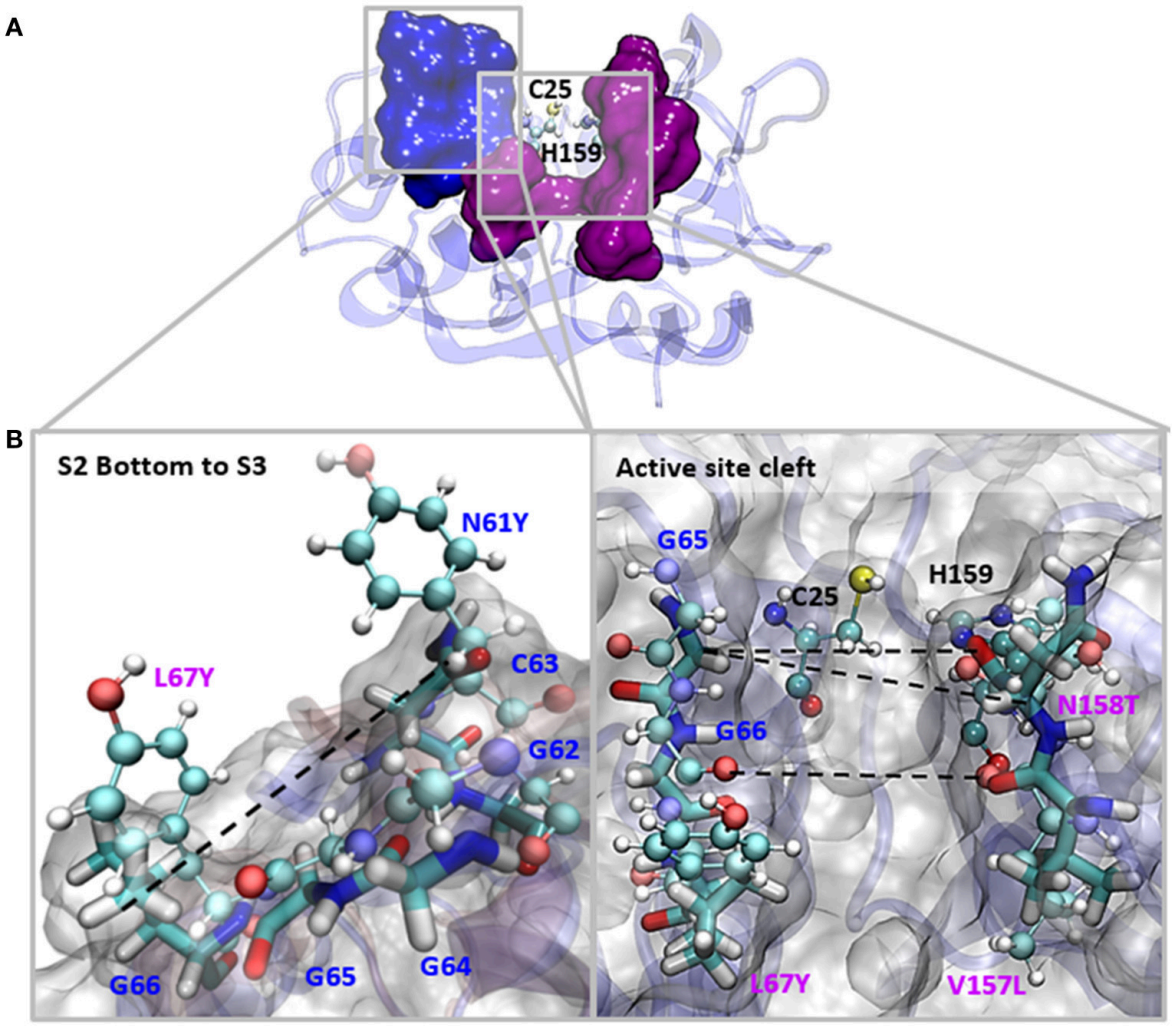
Interatomic distances between residues in the S_2_ and S_3_ sites in MD simulations. **(A)** The most representative structure for *Fh*CL1 is depicted in blue new cartoons, in ball and sticks are depicted Cys25 and His159; blue surface represents the S_3_ residues and pink surface represents the S_2_ residues. **(B)** We zoomed the regions in the squares and show the measured distances with dashed lines. Blue labels show the S_3_ residues and pink labels show the S_2_ residues. *Fh*CL1 residues are depicted in sticks and *Fh*CL2 residues are represented in ball and sticks.

The S_2_ site represents the narrowest part of the active site cleft and is considered the major specificity determinant in papain-like proteases (Schechter and Berger, 1967). Besides the residues mutated in this work, a conserved glycine from the S_2_ wall, Gly65, was predicted to be a main contributor to substrate binding (Hernández Alvarez et al., 2015). Hence, we compared the interatomic distances that define the geometry of this pocket, measuring the distances between Gly65:CA—Thr158:O, Gly65:CA—Thr158:CB and Gly66:O—Val157:O (Figure 5 and Table 3). We also studied the conformation of the S_3_ subsite, as residue 67 also modulates its architecture, and computed the distances among Asn61:CG—Tyr67:CG (Figure 4 and Table 3). Whereas the S_2_ bottom and S_3_ sites of *Fh*CL2 are more constrained than those of *Fh*CL1, the cleft tends to narrow even more in the *Fh*CL1 mutants (around 1.2 Å and 2 Å shorter than *Fh*CL2; Table 3). This may account for their reduced activity toward the Z-Val-Leu-Lys-AMC substrate. Also, a shortening is seen in the width of the less defined S_3_ pocket when Leu 67 of *Fh*CL1 is replaced by Tyr as in *Fh*CL2 (Table 3). This result supports the experimental data previously seen (Figure 3), because mutants bearing this substitution accept Gly better in S_3_, a characteristic of the *Fh*CL2 enzyme.

**Table 3:** Interatomic distances measured as shown in Figure 5 between selected residues in the S_2_ and S_3_ sites in MD simulations.

**Enzyme**	**Distance 61:CG–67:CG**	**Distance 65:CA–158:O**	**Distance 65:CA–158:CB**	**Distance 66:O–157:O**
*Fh*CL1	11.44 ± 0.09	8.57 ± 0.02	10.16 ± 0.06	9.24 ± 0.01
*Fh*CL1 L67Y	9.81 ± 0.05	7.98 ± 0.01	10.02 ± 0.01	9.18 ± 0.01
*Fh*CL1 V157L	10.41 ± 0.06	7.39 ± 0.01	9.60 ± 0.01	7.38 ± 0.05
*Fh*CL1 N158T	10.02 ± 0.08	7.66 ± 0.02	9.47 ± 0.02	8.58 ± 0.01
*Fh*CL1 L67Y V157L	9.58 ± 0.05	7.73 ± 0.01	9.70 ± 0.02	7.30 ± 0.04
*Fh*CL1 L67Y N158T	9.97 ± 0.06	7.67 ± 0.02	9.82 ± 0.02	8.66 ± 0.02
*Fh*CL1 V157L N158T	10.09 ± 0.10	7.58 ± 0.01	9.89 ± 0.02	7.09 ± 0.01
*Fh*CL1 L67Y V157L N158T	9.80 ± 0.08	8.04 ± 0.01	10.23 ± 0.02	7.82 ± 0.06
*Fh*CL2	9.44 ± 0.10	9.30 ± 0.06	11.23 ± 0.04	8.06 ± 0.01

*All distances are given in ångströms (Å) as mean values (± SD) calculated assuming normal distribution of the values obtained from each frame of the MD*.

Recent studies support the fact that the width of the active site pockets is important for a productive enzyme-substrate interaction. Specifically, the binding of a substrate in the active site of human cathepsin K stabilizes the enzyme in a conformation that has a wider S_2_-S_1_ site, while the free enzyme displays a narrower site that hampers substrate accommodation (Novinec, 2017). Similarly, the reduced width of the S_2_ and S_3_ pockets of *Fh*CL1 mutants hinder the binding of substrates into the active site.

In summary, molecular dynamics simulations explain the weak ability of the *Fh*CL1 mutants to cleave after Pro in P_2_ as their S_2_ pocket configuration does not resemble that of *Fh*CL2.

### Functional implications

Although active site conformation is very similar in the papain-family of enzymes, our results provide additional evidence to previous data (Novinec, 2017) suggesting that minor structural changes affect enzyme specificity. Here, we have focused on the differences in the S_2_ active site pocket, finding that despite its central role, it alone cannot fully explain substrate preferences, as seen for human cathepsins (Lecaille et al., 2007). There are clearly residues outside the active site cleft that modulate its conformation, probably belonging to the so-called “protein sectors.” These are dispersed networks of residues spatially contiguous in the structure of a protein that co-evolve and are implicated in structural and functional properties, for example ligand binding specificity and allosteric communication (McLaughlin et al., 2012). For the papain-like cysteine protease family, a single protein sector was identified which shows a continuous spatial distribution of residues around the active site and extends far throughout both domains of the protein (Novinec et al., 2014). Many, but not all, of the sector residues are conserved between the different liver fluke CLs. Our results suggest that the remaining variable residues outside the S_2_ pocket delineate the architecture of the active site and contribute,via long range dynamic correlation patterns to the correct positioning of the substrate. Further structural predictions corroborated by experimental data could provide evidence for this hypothesis in the future.

## Author contributions

IC, LR, and JT: conceived and designed the mutagenesis experiments; IC, LP, and NP-D: performed the mutagenesis experiments and kinetic measurements; FF and AM: performed the molecular dynamics simulations; IC, AO, KZ, and TB: performed the MSP-MS and the PSSCL experiments; IC, AM, JM, CRC, CSC, and JT: contributed reagents, materials and analysis tools; IC, FF, AM, AO, CRC, LR, and JT: analyzed and interpreted the data; IC and JT: wrote the paper.

### Conflict of interest statement

The authors declare that the research was conducted in the absence of any commercial or financial relationships that could be construed as a potential conflict of interest.
