# Supplementary material for: Substrate Specificity of Cysteine Proteases Beyond the S2 Pocket: Mutagenesis and Molecular Dynamics Investigation of Fasciola hepatica Cathepsins L
Source: Front Mol Biosci. 2018 Apr 19;5:40. doi: 10.3389/fmolb.2018.00040 (PMC5917446; doi:10.3389/fmolb.2018.00040)
Supplement: Supplementary file 1 [file Table1.DOCX]

| ***Mutant Enzyme*** | ***Plasmid Template*** | ***Oligonucleotides*** | ***Sequence*** |
| --- | --- | --- | --- |
| *Fh*CL1 L67Y | *Fh*CL1 wild type | *Fh*CL1 L67Y Fw | GGTTGCGGTGGTGGA***TAT***ATGGAAAATGCTTACC |
|  |  | *Fh*CL1 L67Y Rv | GGTAAGCATTTTCCAT***ATA***TCCACCACCGCAACC |
| *Fh*CL1 V157L | *Fh*CL1 wild type | *Fh*CL1 V157L Fw | GTTCACCGCTTCGT***TTG***AACCATGCAGTCTTGG |
|  |  | *Fh*CL1 V157L Rv | CCAAGACTGCATGGTT***CAA***ACGAAGCGGTGAAC |
| *Fh*CL1 N158T | *Fh*CL1 wild type | *Fh*CL1 N158T Fw | CACCGCTTCGTGTG***ACC***CATGCAGTCTTGG |
|  |  | *Fh*CL1 N158T Rv | CCAAGACTGCATG***GGT***CACACGAAGCGGTG |
| *Fh*CL1 L67Y V157L | *Fh*CL1 L67Y | *Fh*CL1 V157L Fw & Rv |  |
| *Fh*CL1 L67Y N158T | *Fh*CL1 L67Y | *Fh*CL1 N158T Fw & Rv |  |
| *Fh*CL1 V157L N158T | *Fh*CL1 wild type | *Fh*CL1 V157L N158T Fw | GTTCACCGCTTCGT***TTGACC***CATGCAGTCTTGGCTG |
|  |  | *Fh*CL1 V157L N158T Rv | CAGCCAAGACTGCATG***GGTCAA***ACGAAGCGGTGAAC |
| *Fh*CL1 L67Y V157L N158T | *Fh*CL1 V157L N158T | *Fh*CL1 L67Y Fw & Rv |  |

**Supplementary Table 1. *Fh*CL1 enzyme variants generated by site-directed mutagenesis.** The amino acid changes introduced into the S_2_ active site pocket of *Fh*CL1 are listed on the first column (papain numbering), the plasmid templates and the oligonucleotide pairs used in the PCR reaction are named in the second and third columns, respectively, and the oligonucleotides sequences are shown in the last column. The nucleotides introducing the desired mutation are highlighted in bold italics.
