# Supplementary material for: Substrate Specificity of Cysteine Proteases Beyond the S2 Pocket: Mutagenesis and Molecular Dynamics Investigation of Fasciola hepatica Cathepsins L
Source: Front Mol Biosci. 2018 Apr 19;5:40. doi: 10.3389/fmolb.2018.00040 (PMC5917446; doi:10.3389/fmolb.2018.00040)
Supplement: Supplementary file 2 [file Table2.pdf]

Supplementary Table 2. *Fh*CL1 hydrolysis of Z-Val-Leu-Lys-AMC peptide substrate, data fit to the Michelis-Menten equation. The table in the second page shows the substrate concentration (X) and initial velocity values (Y, measured in μM AMC released per second), used to determine the K_M and V_{max} parameters for the hydrolysis of Z-Val-Leu-Lys-AMC by *Fh*CL1 (enzyme concentration is 3.17 nM). The third page shows the estimated parameters K_M and V_{max} with the standard errors and the statistics of the data fit calculated by the OriginPro 6.1 software. In the last page the graph of initial velocity vs substrate concentration is showing the data points fitting to the Michaelis Menten equation.

	A(X)	B(Y)
Long Name	Substrate	Vi
Units	uM	uM/s
Comments	VLK	AMC/s
1	1	0,00191
2	2,5	0,00232
3	5	0,00361
4	7,5	0,00461
5	10	0,00599
6	15	0,00626
7	20	0,0063
8	30	0,0065
9	40	0,007
10	60	0,0069
11	120	0,0068
12	200	0,0069

1 *NL Fit (MichaelisM (User)) (19/03/2018 12:11:48)*

Input Data

	Dep/Indep	Data	Range	Weight Type
x	Indep	[Book1]Sheet1!Su	[1*:12*]	
y	Dep	[Book1]Sheet1!Vi	[1*:12*]	No Weighting

Parameters

		Value	Standard Error
B	Vmax	0,00737	2,28984E-4
	Km	3,93734	0,5961

Iterations Performed = 3
Total Iterations in Session = 3
Fit converged - Chi-sqr no longer changed.

Statistics

	Vi
Number of Points	12
Degrees of Freedom	10
Reduced Chi-Sqr	1,7776E-7
Residual Sum of Squares	1,7776E-6
Adj. R-Square	0,94802
Fit Status	Succeeded(101)

Fit Status Code :
101 : Fit converged

Summary

	Vmax		Km		Statistics	
	Value	Error	Value	Error	Reduced Chi-Sqr	Adj. R-Square
B	0,00737	2,28984E-4	3,93734	0,5961	1,7776E-7	0,94802

ANOVA

		DF	Sum of Squares	Mean Square	F Value	Prob>F
Vi	Regression	2	3,89005E-4	1,94502E-4	1094,18826	1,50497E-11
	Residual	10	1,7776E-6	1,7776E-7		
	Uncorrected Total	12	3,90782E-4			
	Corrected Total	11	3,76149E-5			

Fitted Curves Plot

Vi

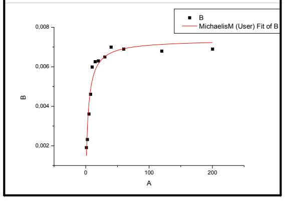

Residual vs. Independent Plot

Vi

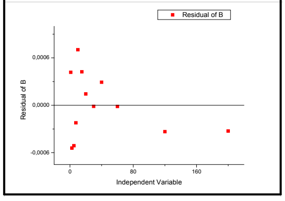

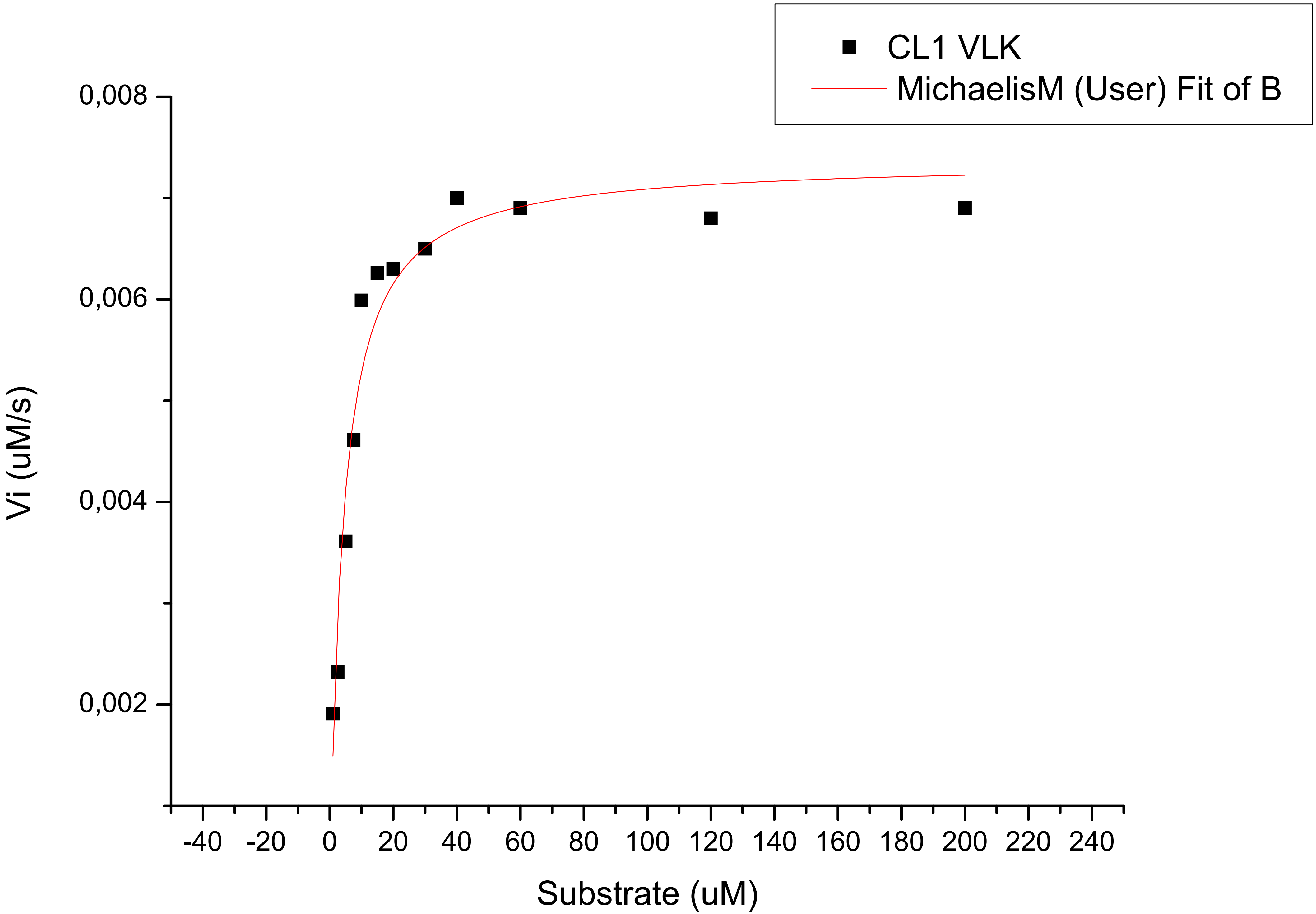
CL1 VLK
MichaelisM (User) Fit of B
0,008
0,006
0,004
0,002
Vi (uM/s)
-40
-20
0
20
40
60
80
100
120
140
160
180
200
220
240
Substrate (uM)
