# Supplementary material for: Substrate Specificity of Cysteine Proteases Beyond the S2 Pocket: Mutagenesis and Molecular Dynamics Investigation of Fasciola hepatica Cathepsins L
Source: Front Mol Biosci. 2018 Apr 19;5:40. doi: 10.3389/fmolb.2018.00040 (PMC5917446; doi:10.3389/fmolb.2018.00040)
Supplement: Supplementary file 3 [file Image1.PDF]

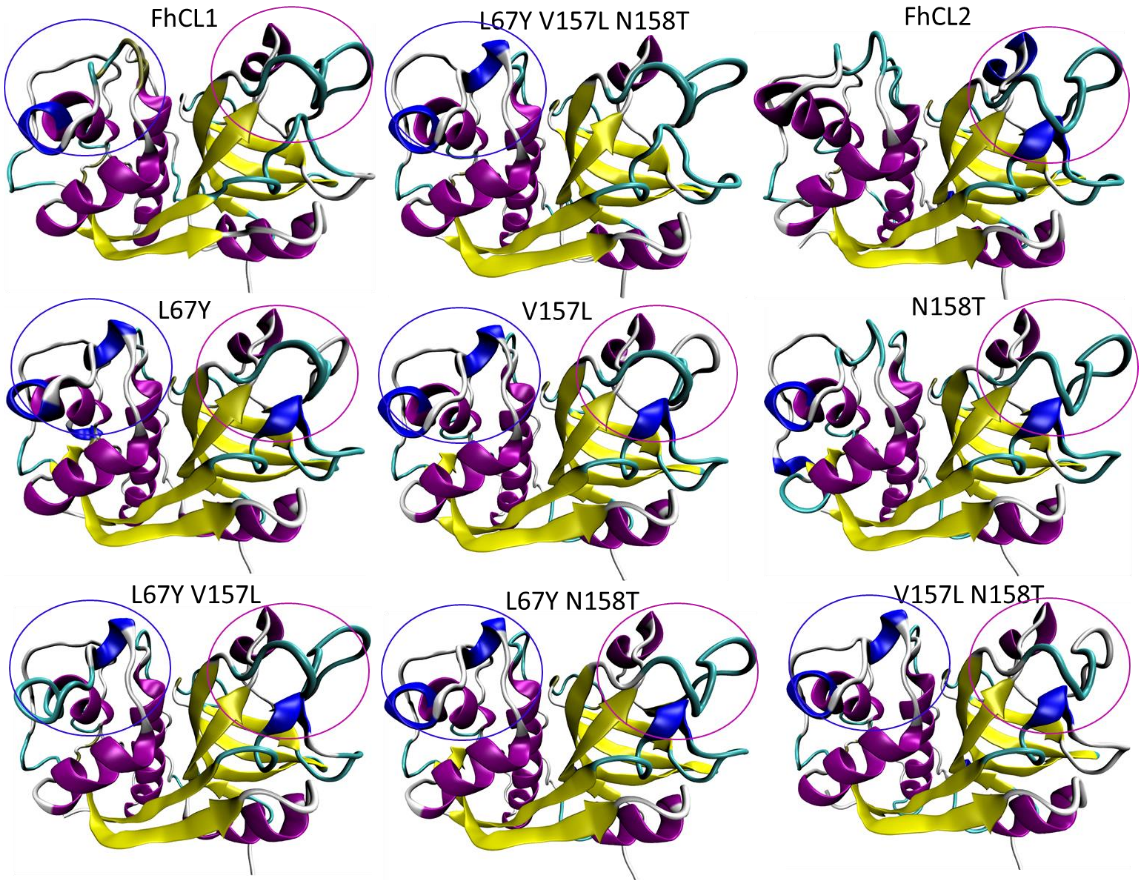

Supplementary Figure 1. Secondary structure of *Fh*CL1, *Fh*CL2 and the mutants. Representative structures of the enzymes from the MD simulation are depicted in new cartoon and colored by secondary structure. Alfa-helices are colored in purple and blue, beta-sheets are colored in yellow, and turns and loops in cyan. The purple and blue circles indicate regions of structural variation between the native and mutant enzymes around the S_2 and S_3 sites, respectively.
